# Phylogenetic analysis, metabolic profiling, and environmental adaptation of strain LCG007: a novel Rhodobacteraceae isolated from the East China Sea intertidal zone

**DOI:** 10.3389/fmicb.2024.1533195

**Published:** 2025-01-07

**Authors:** Cuizhu Liang, Jiahua Wang, Jie Liu, Zekai Wang, Junwei Cao, Xi Yu, Li Zhang, Jiasong Fang

**Affiliations:** ^1^Shanghai Engineering Center of Hadal Science and Technology, College of Marine Sciences, Shanghai Ocean University, Shanghai, China; ^2^Laboratory for Marine Biology and Biotechnology, Qingdao National Laboratory for Marine Science and Technology, Qingdao, China

**Keywords:** intertidal zone, RCA cluster, biogeochemical cycling, environmental adaptations, metabolic versatility

## Abstract

Strain LCG007, isolated from Lu Chao Harbor's intertidal water, phylogenetically represents a novel genus within the family Rhodobacteraceae. Metabolically, it possesses a wide array of amino acid metabolic genes that enable it to thrive on both amino acids or peptides. Also, it could hydrolyze peptides containing D-amino acids, highlighting its potential role in the cycling of refractory organic matter. Moreover, strain LCG007 could utilize various carbohydrates, including mannopine and D-apiose—compounds primarily derived from terrestrial plants—demonstrating its capacity to degrade terrestrial organic matter. It could assimilate ammonia, nitrate and nitrite, and utilizes organic nitrogen sources such as polyamines, along with diverse organic and inorganic phosphorus and sulfur sources. Importantly, unlike very limited *Sulfitobacter* species that possess photosynthetic genes, the genomes of strain LCG007-affiliated genus and all *Roseobacter* species harbor photosynthetic gene clusters. This conservation was further supported by the significant impact of light on the growth and cell aggregation of strain LCG007, suggesting that acquirement of photosynthetic genes could play a crucial role in the speciation of their common ancestor. In terms of environmental adaptability, the genes that encode for DNA photolyase, heat and cold shock proteins, and enzymes responsible for scavenging reactive oxygen species, along with those involved in the uptake and biosynthesis of osmoprotectants such as betaine, γ-aminobutyric acid (GABA), and trehalose collectively enable strain LCG007 to survive in the dynamic and complex intertidal zone environment. Besides, the capacity in biofilm formation is crucial for its survival under conditions of oligotrophy or high salinity. This study enhances our comprehension of the microbial taxonomy within the *Roseobacter* clade affiliated cluster, their survival strategies in intertidal ecosystems, and underscores the significance of their role in nutrient cycling. It also highlights the crucial importance of photosynthetic metabolism for the speciation of marine bacteria and their ecological resilience.

## 1 Introduction

The intertidal zone, commonly known as the littoral zone, is a highly dynamic and complex environment where the interplay of various abiotic and biotic factors creates a challenging setting for microbial life (Otto Ortega-Morales et al., 2010). The continuous exposure to tidal fluctuations subjects these microorganisms to rapid changes in salinity, temperature, and oxygen levels, as well as to physical stressors such as desiccation and wave action (Tee et al., 2021). The upper regions of the intertidal zone are frequently subjected to intense sunlight, which, besides causing DNA damage in microorganisms due to ultraviolet radiation, also leads to rapid increases in temperature and salinity of the sediment, causing the microorganisms to lose water. Conversely, the lower regions may experience prolonged darkness, affecting the photosynthetic capabilities of certain microbes (Alberti et al., 2017). Furthermore, the intertidal environment is also characterized by a gradient of nutrient availability, with some areas being nutrient-rich due to runoff and others being nutrient-poor due to the constant washing away of materials by tides. The complexity of the intertidal environment exerts selective pressures on microbial communities, potentially leading to the evolution of unique adaptations and the development of specialized metabolic functions. Understanding these metabolic adaptations can provide insights into the resilience of microbial life under harsh conditions.

The *Roseobacter* clade affiliated (RCA) cluster, a taxonomically coherent subset within the class *Alphaproteobacteria*, encompasses a collection of marine bacteria with significant ecological implications. Initially observed in the 1970s and 1980s for their characteristic rose-colored pigmentation, these bacteria were enigmatic in terms of their phylogenetic affiliations and ecological roles. The seminal work of Tomoichi Shiba in the early 1990s marked a significant milestone with the first isolation of *Roseobacter denitrificans* OCh 114 and *Roseobacter litoralis* OCh 149, facilitating the taxonomic categorization and ecological appraisal of the RCA cluster (Shiba and Microbiology, 1991). To date, the RCA cluster comprises over 40 distinct bacterial genera, manifesting their ubiquity and numerical dominance in marine ecosystems. Environmentally, the RCA cluster is one of the most abundant marine bacterial groups, comprising 3%−5% of bacteria cells in open ocean surface waters and up to 20% in coastal waters, underscoring their adaptability and indispensable ecological functions within marine ecosystems (Wagner-Döbler and Biebl, 2006; Brinkhoff et al., 2008; Lam-Tung et al., 2015)

Metabolically, RCA species exhibit versatile capabilities, and are known for their capacity to degrade complex organic matter, allowing them to adapt to various environmental conditions. For instance, Sheppard et al. (2012) identified significant presence of *Roseobacter* lineage in marine waters contaminated with petroleum. Moreover, Brito et al. (2006) isolated eight *Roseobacter* strains in Brazilian mangrove sediments, rich in pyrene, naphthalene, and fluoranthene, exhibiting variable aromatic degradation rates (10%–100%). Besides, six *Roseobacter* isolates were screened for the presence of protocatechuate 3,4-dioxygenase, a key enzyme in the β-ketoadipate pathway, and were capable of growth on at least three of the eight aromatic monomers presented (anthranilate, benzoate, p-hydroxybenzoate, salicylate, vanillate, ferulate, protocatechuate, and coumarate) (Buchan et al., 2000, 2001). Additionally, *Roseobacter* lineages can degrade dimethylsulfoniopropionate (DMSP) released by algae through both direct cleavage and demethylation, resulting in the production of dimethyl sulfur (DMS) gas, which plays a crucial role in climate regulation (Dickschat et al., 2010). Importantly, many of the RCA species were reported capable of performing aerobic anoxygenic photosynthesis, allowing them to capture light energy for growth without releasing oxygen (Voget et al., 2015). Such metabolic flexibility makes them an essential component of the marine microbial community which contribute to the biogeochemical cycling of carbon and other nutrients in the ocean.

However, due to high 16S rRNA sequence similarities of over 97% among multiple genera within the RCA cluster, many species may have been misclassified, indicating a need for further taxonomic clarification and reevaluation to accurately reflect the true diversity and ecological roles of these genera. Moreover, although the RCA cluster is predominantly found in nearshore environments (Wagner-Döbler and Biebl, 2006; Brinkhoff et al., 2008), within the intertidal zone—one of the most variable and complex nearshore marine environments—both the ecological functions and adaptive mechanisms of the RCA cluster have received limited attention. This gap in knowledge underscores the necessity for further research to elucidate their roles and strategies for navigating the distinctive environmental challenges inherent to this dynamic ecosystem.

In this study, we utilized an oligotrophic culture medium and photoirradiation to enrich for microbes from the intertidal zone seawater. This approach led to the successful isolation of strain LCG007 within the RCA cluster. Then, we conducted an in-depth investigation of its metabolic traits, and explored the ecological significance and adaptive mechanisms of strain LCG007 to the intertidal environment through comprehensive genomic comparison, metabolic network reconstruction, and biological assays. Additionally, we examined the distribution of photosynthetic genes within strain LCG007 and phylogenetically related species, and further provided evidence for the critical role of photosynthesis in the survival of strain LCG007, particularly under oligotrophic conditions. This research enhances our understanding of microbial diversity and adaptability in the intertidal zone, with broader implications for ecological and biogeochemical processes, particularly in the context of terrestrial organic matter degradation.

## 2 Materials and methods

### 2.1 Enrichment and isolation of the strain

The water sample was collected from the intertidal zone in the East China Sea (38.46°N, 121.84°E) in October 2022, through gently pressing down on the intertidal sediment using a sterile 50 ml centrifuge tube immediately after the tide receded. Then, the sample was diluted to a rate of 1:1,000 in the oligotrophic culture medium, which consisted of artificial seawater (ASM-1) supplemented with 0.2% (v/v) 2-aminoethylphosphonic acid (2-AEP). After a 3-week incubation period at 28°C, the microbial enrichment cultures were diluted again at a ratio of 1:1,000 using ASM-1 medium and were inoculated onto Marine Broth 2216E (MB 2216E)-agar plates at 28°C for 2 weeks. Subsequently, the colonies were isolated and purified through the method of streaking. The isolated strain, designated as strain LCG007 (= MCCC 1K09537), was expanded in MB 2216E liquid medium under aerobic conditions at 28°C, then aliquoted and stored at −80°C in the same medium with the addition of 20% (v/v) glycerol.

The ASM-1 used in this study consists of NaCl (28 g/L), CaCl_2_·2H_2_O (1.5 g/L), MgCl_2_·6H_2_O (5.5 g/L), MgSO_4_·7H_2_O (0.7 g/L), KCl (0.7 g/L), NaHCO_3_ (0.5 g/L). The ASM-1 was autoclaved and supplemented with 1:1,000 trace elements, including FeCl3·6H_2_O (30 mg/L), MnCl_2_·4H_2_O (2 mg/L), ZnSO_4_·7H_2_O (0.23 mg/L), CoCl_2_·6H_2_O (0.2 mg/L), Na_2_MoO_4_·2H_2_O (0.1 mg/L), Na_2_SeO_3_ (0.2 mg/L) and NiCl_2_·6H_2_O (0.2 mg/L). The supplementary vitamin mixture included thiamine (1.8 mg/L), nicotinic acid (0.0984 mg/L), pantothenic acid (0.0984 mg/L), pyridoxine (0.1 mg/L), biotin (0.0001 mg/L), folate (0.001 mg/L), cobalamin (0.001 mg/L), myo-inosito (0.001 mg/L) and 4-aminobenzoic acid (0.08 mg/L; final concentration). Trace elements and vitamin mixture were filtered through 0.1 μm filters before usage. The MB 2216E medium was prepared by adding 5 g of peptone and 1 g of yeast extract to each liter of ASM-1, followed by adjusting pH to a range of 7.5 to 7.6. After autoclaving, the aforementioned trace elements and vitamins were supplemented.

### 2.2 Morphology and physiology analysis

Morphological observations of strain LCG007 was performed via a transmission electron microscopy (JEM-1400, JEOL, Tokyo, Japan) with cultures grown in MB 2216E liquid medium at 28°C for 1 day. The Gram stain was conducted with a Gram-staining kit (Hangzhou Tianhe Microbiological Reagent Co., Ltd., Hangzhou, China). Enzyme activities were determined using the API ZYM system (bioMérieux).

For cellular fatty acid analysis, strain LCG007 was cultured in MB 2216E at 28°C for 3 days. The fatty acids were saponified, methylated, and extracted following the standard protocol of MIDI (Shorrock Microbial Identification System, version 6.0). Fatty acid methyl esters were analyzed by gas chromatography using the Agilent Technologies 6850 and identified using the RTSBA6.0 database of the Microbial Identification System (Athalye et al., 1985). For the examination of polar lipids in strain LCG007, polar lipids were extracted and isolated on a silica gel 60 F254 aluminum-backed thin layer chromatography plate (10 × 10 cm; Merk 5554), followed by further analysis introduced by Minnikin et al. (1984). Respiratory quinones were extracted using the method described by Minnikin et al. (1984) and analyzed by HPLC, as detailed by Tindall (1990). The chromatographic plates were developed using a two-dimensional solvent system. In the first dimension, a solvent mixture of chloroform, methanol, and water was utilized in a volumetric ratio of 65:24:4. The second dimension employed a mixture of chloroform, glacial acetic acid, methanol, and water in a volumetric ratio of 80:15:12:4. After development, the plates were sprayed with a 5% (w/v) solution of phosphomolybdic acid in alcohol and subsequently heated at 160°C for 10–15 min to visualize the total lipid content.

### 2.3 Determining the range of temperature, salinity, and pH required for growth of strain LCG007

To identify the optimal growth conditions of strain LCG007, we tested the temperature range by assessing its growth at 5, 10, 15, 20, 28, 35, and 40°C. We measured the salinity range for growth under conditions with NaCl concentrations of 0%, 1%, 2%, 3%, 4%, 5%, and 6% (w/v) at a constant temperature of 28°C. Furthermore, we established the pH range for growth by evaluating its growth at pH levels of 4, 5, 6, 7, 8, 10, and 11 using HCl or NaOH. Subsequently, the optical density at 600 nm (OD_600_) was measured using a spectrophotometer. All experiments were conducted with three replicates.

### 2.4 Microbial utilization of different substrates

The strain LCG007 was characterized using the Biolog GEN III microplate (Biolog Inc., Hayward, CA, USA), which includes 95 different carbon substrates. Furthermore, the capacity of strain LCG007 to metabolize various substrates was assessed through cultivation experiments in ASM-1 enriched with the following substrates as the sole carbon source: peptone, D-alanine, D-phenylalanine, D-lysine, D-glutamate, peptidoglycan, D-apiose, sodium malate, sodium benzoate, sodium citrate, sodium acetate, polyhydroxybutyrate, urea, hypoxanthine, xanthine, casein, tyrosine. The concentrations of casein, tyrosine, xanthine, hypoxanthine, and peptone were 0.5 g/L, and those of other carbohydrates were 5 mM. In detail, strain LCG007 was cultivated in MB 2216E medium to the logarithmic phase (OD_600_ = 0.4), and then washed and resuspended three times with a 10-fold amount of artificial seawater. Then, a small amount of the resuspended bacterial suspension was added to culture flasks containing these substrates for cultivation at 28°C. On day 0 (before cultivation) and day 7 (after cultivation), a small amount of the bacterial culture broth was diluted 10^4^-fold and spread on plates containing MB 2216E medium, followed by incubation at 28°C for 3–5 days. All these experiments were conducted with three replicates.

### 2.5 Effect of visible light on strain LCG007 growing dynamics

To investigate the impact of visible light on the growth kinetics of strain LCG007, cultures in the logarithmic phase were transferred into oligotrophic medium containing 1% MB 2216E organic compounds and incubated at 28°C. The light group received 12 h of light exposure daily, while the dark group was wrapped in aluminum foil to maintain a light-free condition. After 72 h of incubation, the biofilm formed at the bottom of the culture flask was stained using the SYTO 9/PI live/dead bacterial dual stain kit and then observed under a fluorescence microscope. All experiments were conducted with three replicates.

### 2.6 Detection of biofilm formation

To further investigate the bacterial biofilm, we diluted the strain LCG007 during the logarithmic growth phase with 2216E medium to an optical density (OD_600_) of 0.3. Then, 10 μl of the diluted bacterial suspension was transferred along with 190 μl of either MB 2216E or oligotrophic medium into three wells of two 96-well microtiter plates. The plate in the light group were left untreated, while the plate in the dark group were wrapped in aluminum foil. Then, these plates were placed in a constant temperature incubator (at 28°C). After a 3-day incubation, we carefully aspirated the bacterial supernatant and gently washed the culture wells with ddH_2_O three times to remove non-adherent bacterial cells. Then, the wells were dried at room temperature for 10 min, and 250 μl of a 1% (w/v) crystal violet solution was added to each well. After 25 min, the crystal violet solution was removed, the plates were carefully washed with ddH_2_O three times, and the wells were dried at room temperature for 10 min. Finally, photographs of these wells were taken and the color intensity was compared to assess the biofilm formation.

### 2.7 Genomic DNA extraction, sequencing and assembly

The genomic DNA of strain LCG007 was extracted using the DNA Extraction Kit (Tiangen Biotech Co., Ltd., Beijing, China). The strain's genome sequencing was carried out by MajorBio (Shanghai Majorbio Bio-pharm Technology Co., Ltd., Shanghai, China), employing a combination of PacBio RS II Single Molecule Real-Time (SMRT) technology (Pacific Biosciences, Menlo Park, CA, USA) and the Illumina HiSeq 2500 sequencing platform (Illumina Inc., San Diego, CA, USA).

For Illumina sequencing, about 1 μg of genomic DNA was fragmented into 400–500 bp pieces using a Covaris M220 Focused Acoustic Shearer (Covaris, Woburn, MA, USA), following the manufacturer's guidelines. These fragments were then used to prepare Illumina sequencing libraries with the NEXTFLEX Rapid DNA-Seq kit (NEXTFLEX, San Jose, CA, USA).

Regarding Pacific Biosciences sequencing, 15 μg of DNA was dispensed and centrifuged in a Covaris g-TUBE at 6000 RPM for 60 s using an Eppendorf 5424 centrifuge (Eppendorf, Hamburg, Germany). The DNA fragments were purified, end-repaired, and ligated with SMRTbell adapters following Pacific Biosciences' protocols. The library was purified three times using 0.45 × volume of Agencourt AMPure XP beads (Agencourt, Scottsdale, AZ, USA), according to the manufacturer's recommendations. A library with an insert size of ~10 kb was prepared and then sequenced in a SMRT cell using standard procedures.

Bioinformatics analysis was conducted using data from both PacBio and Illumina sequencing platforms. The raw image data were processed into sequence data through base calling, yielding raw reads that were stored in FASTQ format. Quality trimming was applied to remove poor-quality sequences, and quality control statistics were generated. The reads were assembled into contigs using the hierarchical genome assembly process (HGAP) and Canu software (Koren et al., 2017). The final circularization was manually checked and completed, resulting in a chromosome and a plasmid representing the complete genome. Subsequently, the PacBio assembly was polished using Illumina reads with the Pilon tool (Walker et al., 2014). The complete genome sequence of strain LCG007 was submitted to the RefSeq database under accession number GCF_040801785.1.

### 2.8 Gene annotation and genomic comparison

The prediction and annotation of open reading frames (ORFs) were performed by NCBI Prokaryotic Genome Annotation Pipeline (PGAP) (Tatusova et al., 2016). The predicted protein sequences were also subjected to alignment against the Clusters of Orthologous Groups of proteins (COG) (Galperin et al., 2021) and TransporterDB 2.0 (Elbourne et al., 2017) databases employing the BLASTp algorithm (version 2.9.0) with the following parameters set for significance: an identity threshold of 50%, a query coverage of 80%, an e-value cutoff of 1 × 10^−5^, and a minimum score of 40. Additionally, BlastKOALA (Kanehisa et al., 2016) was applied to facilitate the assignment of Kyoto Encyclopedia of Genes and Genomes (KEGG) functional annotations. IslandViewer 4 was engaged to prognosticate the existence and precise positioning of genomic islands embedded within the genomic sequence (Bertelli et al., 2017).

To classify the protein families of strain LCG007 alongside its phylogenetically related strains, we utilized OrthoMCL (version 2.0.9) (Bertelli et al., 2017) with following parameters: sequence identity of 50%, query coverage of 50%, *E*-value threshold of 1 × 10^−10^, a minimum score of 40, and MCL inflation value set at 1.5. The protein families identified exclusively in a single strain were classified as strain-specific. The average nucleotide identity (ANI) and average amino acid identity (AAI) were calculated using FastANI (Jain et al., 2018) and CompareM (https://github.com/dparks1134/CompareM, accessed on 10 December 2023), respectively, adhering to their standard parameter settings.

### 2.9 Phylogenetic analysis

The taxonomic identification of strains LCG007, D35 and HSMS-29 was initially using GTDB-Tk toolkit (reference database version: Release 07-RS207) (Chaumeil et al., 2020). Moreover, we employed the method of POGO-DB (Lan et al., 2014) to further elucidate the phylogenetic positions of strain LCG007. In detail, the protein sequences of the 73 globally-conserved marker genes were identified from each genome based on their COG annotation (listed in http://pogo.ece.drexel.edu/about.php). Then, these protein sequences were aligned individually using Clustal Omega (Sievers and Higgins, 2021), and the positions that had gaps (“–”) in more than 50% of the alignments were removed. Subsequently, the degaped alignments were concatenated, and was used to construct a phylogenetic tree using IQ-TREE (Lam-Tung et al., 2015) with a bootstrap test with 1,000 iterations. Finally, these phylogenetic trees were visualized using the Interactive Tree Of Life (iTOL) online server (Letunic and Bork, 2021).

## 3 Results and discussion

### 3.1 Description of strain LCG007

**Figure 1:**
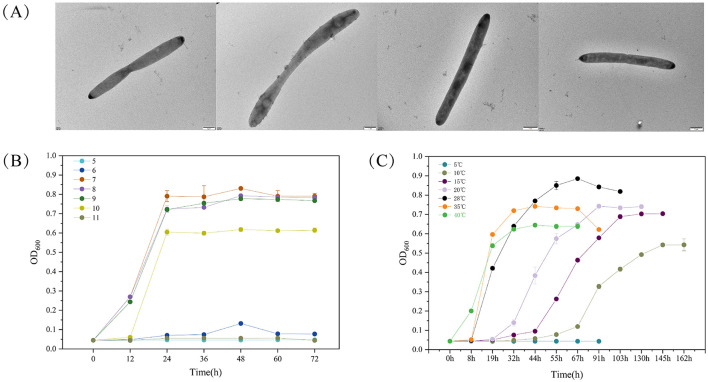
The transmission electron micrograph of strain LCG007 **(A)**. The growth curve of strain LCG007 under multiple temperatures **(B)**, and at different pH **(C)**. Each experiment has three biological replicates.

We utilized an oligotrophic culture medium and photoirradiation to selectively enrich microbes from the intertidal seawater of Lu Chao Harbor along the East China Sea, and strain LCG007 was successfully isolated. Examination via transmission electron microscopy (TEM) showed that the cellular dimensions of strain LCG007 are rod-shaped, measuring 0.8–1.1 μm in width and 2.0–9.0 μm in length, with no distinct flagella detected (Figure 1). Incubation on MB 2216E-agar plates at 28°C, the colonies appeared circular, smooth, convex, and yellowish white, with diameters ranging from 1.5 to 2.5 mm. The strain exhibited growth at temperatures ranging from 5 to 40°C, with an optimal growth temperature of 35°C (Figure 1). Its growth requires a pH range of 6–8, with an optimal pH of 7 (Figure 1).

The respiratory quinone of strain LCG007 was identified as ubiquinone-10 (Q-10) (Supplementary Figure S1). Additionally, for strain LCG007, significant proportions (>1%) of C_16:0_ (15.06%), C_17:0_ (1.86%), C_18:0_ (3.53%), C_20:0_ (2.43%), C_18:1_ ω7c (56.54%), 11-methyl C_18:1_ ω7c (2.3%), and C_10:0_ 3-OH (3.2%) were detected (Table 1). The predominant polar lipids detected in strain LCG007 included phosphatidylcholine (PC), phosphatidylethanolamine (PE), phosphatidylglycerol (PG), diphosphatidylglycerol (DPG), phosphoglycolipid (PGL), aminoglycophospholipid (APGL), four aminolipids (AL), seven phospholipids (PL), three glycolipids (GL), and four unidentified lipids (Supplementary Figure S2).

**Table 1:** Cellular fatty acid composition (%) of strain LCG007 and the type strains of other species.

**Fatty acid**	**1**	**2**	**3**	**4**	**5**	**6**	**7**	**8**
**Straight chain**								
C_16:0_	15.06	11.5	1.7	6.6	1.4	2.8	9.6	4.3
C_17:0_	1.86	2.7	–	1.1	–	0.7	–	–
C_18:0_	3.53	1.1	3.4	0.7	1.6	2.8	1.2	1.3
C_20:0_	2.43	–	–	–	–	–	–	–
**Unsaturated**								
C_12:1_ ω7c	–	–	–	–	–	4.0	3.0	2.9
C_18:1_ *ω7c*	56.54	53.5	87.7	85.5	92.4	82.1	74.1	80.7
C_18:2_ ω9,13c	–	–	–	–	–	–	–	2.3
C_18:2_ ω7,13c	–	–	–	–	1.0	2.9	–	–
C_18:1_ ω7c 11-methyl	2.3	15.7	2.0	1.3	–	–	7.1	3.4
**Hydroxy**								
C_10:0_ 3-OH	3.2	2.3	2.6	1.6	2.4	2.8	2.0	3.3
C_12:0_ 3-OH	–	0.6	–	1.5	–	–	1.4	–

Strains: 1, strain LCG007; 2, *Sulfitobacter sabulilitoris* HSMS-29 (Park et al., 2019); 3, *Roseobacter insulae* YSTF-M11 (Lee et al., 2023); 4, *Roseobacter litoralis* OCh 149 (Park et al., 2019); 5, *Roseobacter fucihabitans* B14 (Hahnke et al., 2024); 6, *Roseobacter cerasinus* AI77 (Park et al., 2019); 7, *Roseobacter ponti* MM-7 (Park et al., 2019); 8, *Roseobacter denitrificans* OCh 114 (Park et al., 2019); –, not detected or ≤ 0.5%.

Enzyme activities were assessed using the API ZYM system (bioMérieux) that strain LCG007 exhibited positive results for alkaline phosphatase, acid phosphatase, α-glucosidase, esterase (C4), esterase lipase (C8), valine arylamidase, leucine arylamidase, cysteine arylamidase, α-galactosidase, β-galactosidase, β-glucosidase, N-acetyl-β-glucosaminidase, α-mannosidase, α-fucosidase, and naphthol-AS-BI phosphohydrolase. In contrast, the activities of lipase (C14), trypsin, and α-chymotrypsin, as well as β-glucuronidase, were not detected (Table 2).

**Table 2:** Differential characteristics of strain LCG007 compared to the type strains of other species.

**Characteristic**	**1**	**2**	**3**	**4**	**5**	**6**	**7**	**8**
Temperature range for growth (°C)	5–35	4–30	4–37	2–30	10–28	10–35	4–35	2–30
NaCl concentration range for growth (%, w/v)	1.0–5.0	1.0–2.0	0–5.0	ND	1.0–3.0	1.0–5.0	1.0–5.0	ND
Cell shape	Rod-shaped	Ovoid or rod	Ovoid or rod	Ovoid or rod	Ovoid or rod	Coccoiid to oval	Coccoiid to oval	Ovoid or rod
Cell size (μm)	0.5–1.1 × 2.0–9.0	0.3–1.0 × 0.8–9.0	0.4–1.0 × 0.6–5.0	0.6–0.9 × 1.0–2.0	0.8–1.1 × 1.2–2.9	0.5–0.9 × 0.7–1.4	1.0–1.2 × 1.5–1.8	0.6–0.9 × 1.2–2.0
Colony color	Yellowish white	Yellowish white	Yellowish white	Pink	Beige to pink	Beige to pink	Cream	Pink
**Hydrolysis and utilization of**								
Casein	+	–	–	+	+	–	–	+
Urea	+	ND	–	–	–	– (+)	–	–
Xanthine	+	–	–	+	+	+	+	+
L-tyrosine	+	–	–	+	–	+	+	+
L-glutamate	+	ND	–	+	+	–	–	+
L-arabinose	w	–	–	+	–	–	–	+
D-fructose	w	–	–	+	+	– (+)	–	–
D-galactose	+	ND	+	–	+	– (+)	–	+
D-mannose	+	–	+	+	+	–	–	+
Cellobiose	+	–	+	+	+	–	–	–
Maltose	w	ND	–	–	+	–	–	+
Trehalose	w	ND	+	–	+	–	–	–
Malate	+	–	–	+	+	– (+)	–	+
Acetate	+	+	+	+	+	– (+)	–	+
**Enzyme activity (API ZYM)**								
Alkaline phosphatase	+	+	+	–	+	+	+	+
Esterase (C4)	w	+	+	–	+	+	–	–
Esterase lipase (C8)	w	ND	+	–	+	+	–	–
Valine arylamidase	w	+	–	–	+	–	–	–
Acid phosphatase	+	–	+	+	+	+ (–)	–	+
Naphthol-AS-BI-phosPhohydrolase	+	–	+	–	+	+	–	–
DNA G + C content (mol%)	65.12	65.0	60.3	57.2 ± 0.2	57.55	61.0	60.8	59.6 ± 0.5

Strains: 1, strain LCG007; 2, *Sulfitobacter sabulilitoris* HSMS-29 (Park et al., 2019); 3, *Roseobacter insulae* YSTF-M11 (Lee et al., 2023); 4, *Roseobacter litoralis* OCh 149 (Shiba and Microbiology, 1991; Jung et al., 2017); 5, *Roseobacter fucihabitans* B14 (Hahnke et al., 2024); 6, *Roseobacter cerasinus* AI77 (Muramatsu et al., 2020; Lee et al., 2023); 7, *Roseobacter ponti* MM-7 (Jung et al., 2017); 8, *Roseobacter denitrificans* OCh 114 (Shiba and Microbiology, 1991; Jung et al., 2017); +, Positive; –, negative; w, weak growth; ND, not determined. Data in brackets indicate varying results from different studies.

### 3.2 The phylogeny of strain LCG007

Strain LCG007 exhibited the highest 16S rRNA gene sequence similarity at 98.77% with “*Sulfitobacter*” sp. D35, with lower affinities to type strains *Roseobacter insulae* YSTF-M11^T^ (98.283%), *R. fucihabitans* B14^T^ (97.802%), *Sulfitobacter aestuariivens* TSTF-M16^T^ (97.737%), and “*Sulfitobacter*” *sabulilitoris* HSMS-29^T^ (97.534%). Moreover, the ANI values indicated that strain LCG007 shares the highest similarity at 77.59% with strain D35, followed by strain HSMS-29 at 73.58% and *S. faviae* S5-53^T^ at 72.50% (Supplementary Table S1). These results indicated that strain LCG007 is more closely related to strain D35 than to strain HSMS-29, and this relationship is further supported by the analysis of average amino acid (AAI) identity (Supplementary Table S2). However, the genome-based taxonomic analysis using gtdb-tk toolkit showed that strains LCG007 and HSMS-29 were categorized within genus labeled as “*Sulfitobacter_E*,” while strain D35 was within genus *Roseobacter*.

**Figure 2:**
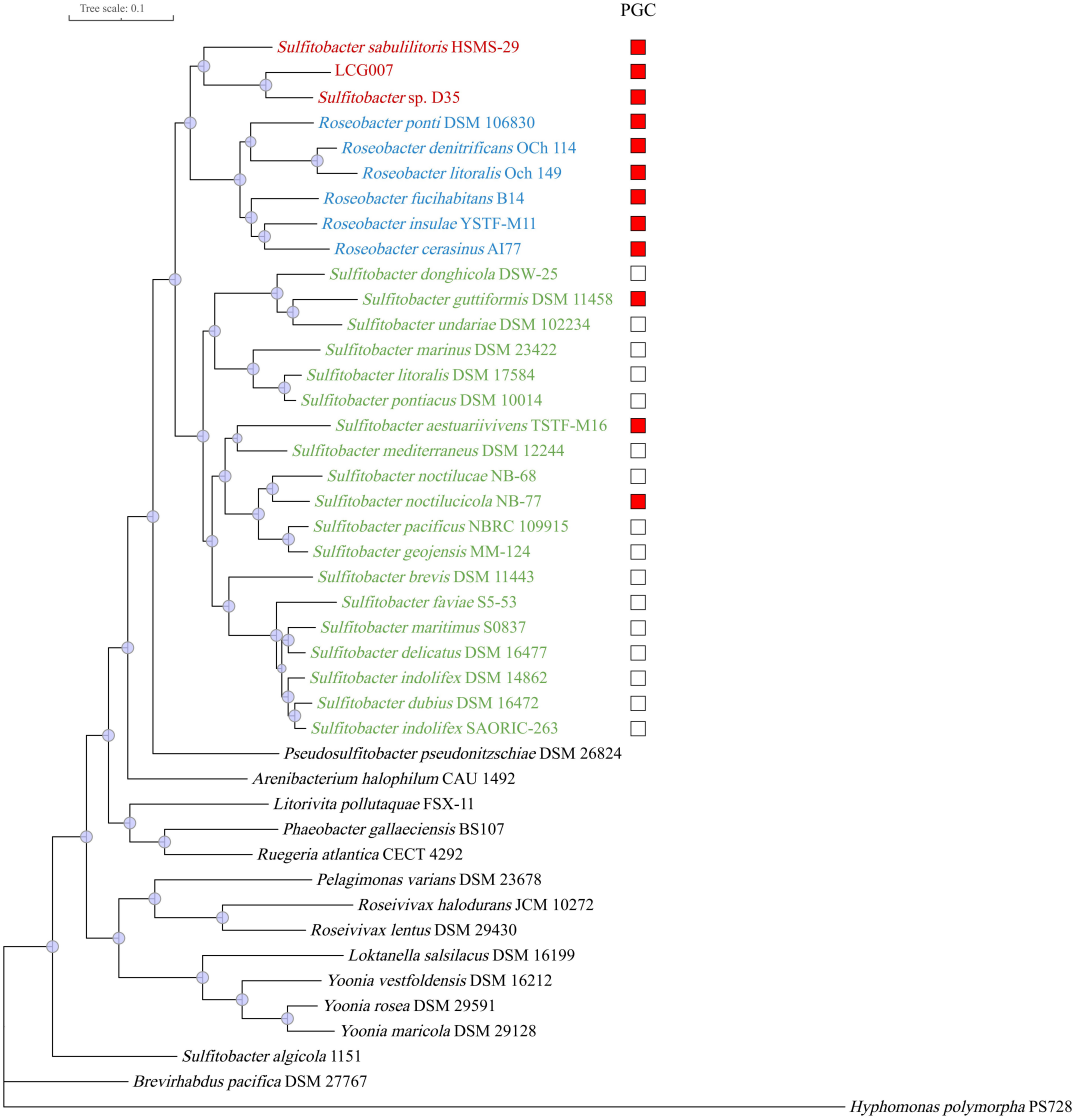
Phylogeny of strain LCG007 and related species based on 73 globally-conserved marker genes. Subtrees of the strain LCG007, *Roseobacter* and *Sulfitobacter* are highlighted with red, blue, green or red, respectively. The presence of the photosynthesis gene cluster (PGC) in the respective genomes is indicated with a red square.

To accurately determine the phylogenies of strains LCG007 and D35, we employed the 73 conserved protein sequences (known as POGO-DB) to construct a phylogenetic tree, encompassing strains LCG007, D35, HSMS-29 and all type strains of the genera *Roseobacter* and *Sulfitobacter* (Figure 2). The results revealed that strains LCG007, together with strains D35 and HSMS-29, formed a unique branch, which is closely related to, but not included within, genus *Roseobacter* (Figure 2).

Moreover, the polar lipid profiles of strain LCG007 and HSMS-29 were distinguishable from those of six other *Roseobacter* species. For example, strain LCG007 and HSMS-29 exhibited high levels of glycolipids, phosphoglycolipids, and aminoglycophospholipids, which were not detected in other *Roseobacter* type strains (Shiba and Microbiology, 1991; Kumari et al., 2016; Jung et al., 2017; Muramatsu et al., 2020; Lee et al., 2023; Hahnke et al., 2024). In addition, strains LCG007 (65.12%) and HSMS-29 (65.0%) exhibit notably higher G+C content compared to the other strains, which display relatively lower G+C content. Furthermore, strains LCG007 and HSMS-29 exhibited a higher C_16:0_ content (>11%) compared to other strains (Park et al., 2019), which showed a relatively lower abundance. Conversely, the C_18:1_ ω7c content (< 57%) in strains LCG007 and HSMS-29 was markedly lower than that observed in the other strains. Besides, fatty acid C_20:0_ (2.43%) was found in significant amounts in strain LCG007, while it was undetectable ( ≤ 0.4%) in the other *Roseobacter* species (Table 1). The collective evidence indicated that both strains LCG007 and D35 belong to a novel genus labeled as “*Sulfitobacter_E*” in GTDB taxonomy.

### 3.3 The genomic features of strain LCG007

The complete genome sequence of strain LCG007 spans a total length of 4,243,571 base pairs, comprising a single chromosome and one plasmid (Table 3). The GC content of this genome is 65.12%. It encodes three ribosomal RNA operons, 42 transfer RNAs and 4,050 predicted protein-coding genes. Among these protein-coding genes, 3,196 (79.35%) are categorized into 23 distinct clusters of orthologous groups (COGs). The most prevalent COG categories are COG-E (involved in amino acid transport and metabolism), COG-G (related to carbohydrate transport and metabolism), and COG-R (assigned to general function prediction only), as detailed in Supplementary Table S3. Furthermore, 13 genomic islands and three prophage regions have been detected within the genome of strain LCG007 (Table 3). Additionally, 796 genes, which constitute up to 19.65% of the coding genes, were found to lack homologs in the genomes of strains D35, HSMS-29, and the six *Roseobacter* type strains (with an BLASTp identity threshold of 50%, query coverage of 50%, and an e-value cutoff of 1e-5). This genetic influx may have provided the necessary variation for the emergence of strain LCG007, highlighting the significance of horizontal gene transfer in microbial evolution and speciation.

**Table 3:** Genome features of strain LCG007.

**Items**	**Description**
Size (bp)	4,243,571
G + C content (%)	65.12
Total genes	3,109
Protein-coding genes	4,050
Genes assigned to COG	3,052
rRNA operons	3
tRNA genes	42
ncRNA genes	3
Pseudogene	25
Gene islands	13
Plasmid	1

### 3.4 The metabolic characteristics of strain LCG007

**Figure 3:**
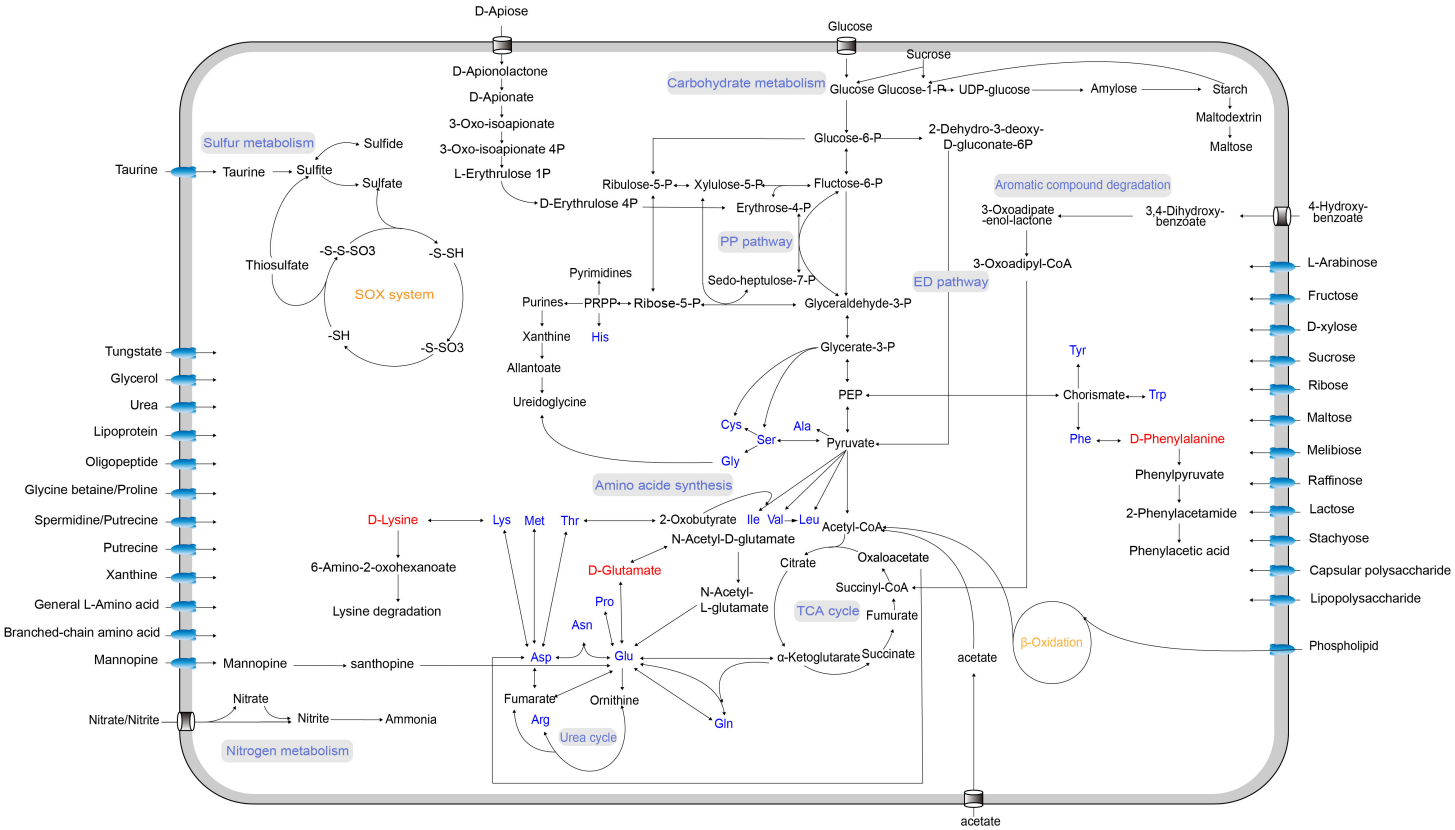
Predicted metabolic pathways of strain LCG007. The arrows indicate one or more reactions. Blue font indicates amino acids, and red font highlights the D-amino acids.

To investigate the metabolic characteristics and ecological potentials of strain LCG007, we reconstructed its metabolic pathways and compared them with those of the strain D35, HSMS-29, and six *Roseobacter* type strains (Figure 3). The metabolism of carbon, nitrogen, sulfur, and phosphorus will be detailed below.

#### 3.4.1 Amino acid metabolism

Strain LCG007 possesses a significant number of 438 genes in the COG category E (amino acid transport and metabolism), accounting for 10.83% of its protein-coding genes. Despite having the metabolic capability to synthesize all 20 essential amino acids, strain LCG007 retains a sophisticated transport system for amino acids and peptides, with a total of 141 associated genes (Supplementary Table S4). This substantial genetic repertoire indicates a considerable dependence on amino acid metabolism for its survival. Additionally, it harbors 10 genes encoding extracellular (signalP-fused) peptidases and could growth using peptone as the sole carbon and nitrogen source, suggesting proteolysis could be an important lifestyle of strain LCG007.

**Figure 4:**
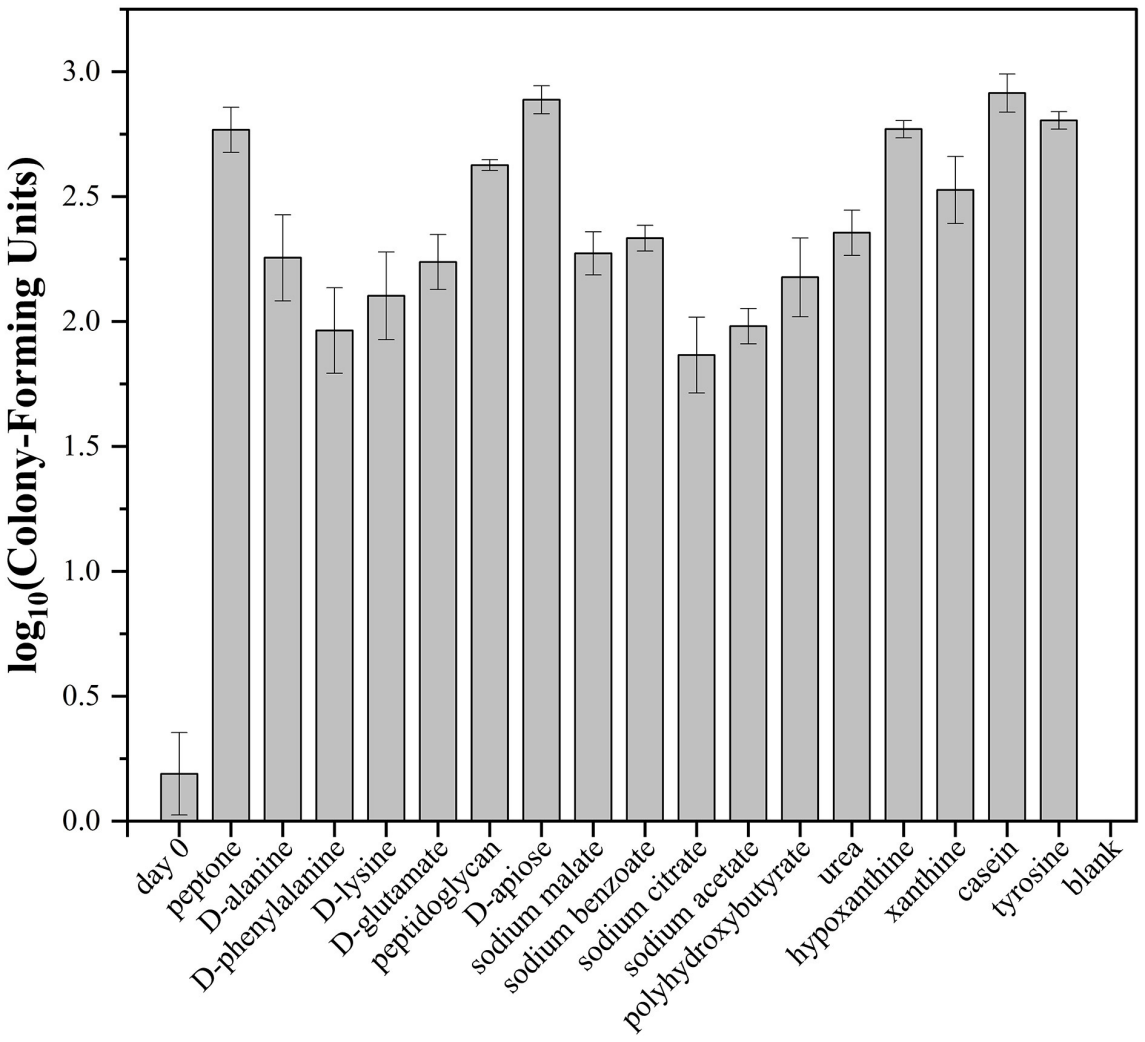
The colony-forming units under different carbohydrates by cell counting. The group designated as “blank” refers to the cultivation medium devoid of any carbon or nitrogen sources. Following a 7-day incubation period, the bacterial broth was spread onto MB 2216E agar plates to enumerate the colony-forming units (CFU). The “day 0” group represents the mean CFU count of the bacterial broth when applied directly to the MB 2216E agar plates without prior cultivation. Each experiment has three biological replicates.

As for D-amino acid metabolism, strain LCG007 possessed the gene of D-amino acid transaminase (EC:2.6.1.21), capable of converting D-phenylalanine into phenylacetic acid, which can be further oxidized by phenylacetic acid degrading pathways (AB1M95_RS08750–AB1M95_RS08805). Besides, this enzyme also converts D-lysine into 6-amino-2-oxohexanoate, which is then metabolized through the lysine degradation pathways. Additionally, strain LCG007 has genes of D-glutamate N-acetyltransferase (EC: 2.3.1.312) and N-acetyl-D-glutamate racemase (EC: 5.1.1.25), which could transform D-glutamate to N-acetyl-L-glutamate. Besides, our experiments further confirmed that strain LCG007 is capable of growing with D-phenylalanine, D-lysine, D-glutamate, D-serine and D-alanine as the sources of carbon and nitrogen (Figure 4, Supplementary Figure S3 and Supplementary Table S6).

Interestingly, the operon encoding a peptide ABC transport system (AB1M95_RS02135–AB1M95_RS02155) also contains a strain-specific gene encoding N-carbamoyl-D-amino-acid hydrolase (EC: 3.5.1.77, AB1M95_RS02160), strongly suggesting that strain LCG007 could intake and hydrolyze D-amino acid-containing peptides intracellularly (Supplementary Table S4). Meanwhile, the identification of four genes encoding extracellular D-alanyl-D-alanine carboxypeptidases in strain LCG007 suggested the extracellular decomposition of D-amino acid-containing components (Supplementary Table S5). We further confirmed that peptidoglycan can act as the sole carbon source for the growth of strain LCG007 (Figure 4 and Supplementary Figure S3). The evidence collectively underscored its potential ecological function in the recycling of recalcitrant organic matter within marine surface waters.

#### 3.4.2 Carbohydrate metabolism

As for carbohydrate metabolism, strain LCG007 is predicted to contain 316 genes within the COG-G, accounting for 7.87% of all its protein-coding genes. Furthermore, we predicted 88 genes involved in transport systems of various carbohydrates, including monosaccharides (e.g., fructose, ribose, mannose, L-arabinose, and D-xylose), disaccharides (e.g., sucrose, melibiose, maltose/maltodextrin, and lactose), oligosaccharides (e.g., raffinose, stachyose, and melibiose) and polyol, as well as numerous non-specific sugar ABC transport systems (Supplementary Table S4). The genes of L-arabinose transporters and metabolic enzymes were only identified in strains LCG007 and D35, suggesting a unique ecological function of them. Experimentally, we confirmed that strain LCG007 could metabolize L-arabinose, D-glucose, D-fructose, glycerol, D-galactose, D-cellobiose, D-mannose, D-trehalose, D-melibiose, D-maltose, D-lactose, and D-gluconate using Biolog GEN III MicroPlates (Supplementary Table S6).

Specifically, D-apiose, a branched-chain pentose sugar found in the cell walls of higher plants, particularly in rhamnogalacturonan II (RG-II) and aquatic monocots, contributes to cell wall stability through ester bonds with boric acid. It is also a key component in many plant secondary metabolites, such as flavonoids, coumarins, and lignans (Molhoj et al., 2003). Strain LCG007 possesses a strain-specific gene cluster (AB1M95_RS14470–AB1M95_RS14495), comprising genes encoding D-apionolactonase (EC:3.1.1.115), D-apiose aehydrogenase (EC:1.1.1.420), D-apionate oxidoisomerase (EC:1.1.1.421), 3-oxoisoapionate kinase (EC:2.7.1.231), and 3-oxoisoapionate-4-phosphate decarboxylase (EC:4.1.1.121), which collectively catalyze the degradation of D-apiose. We further confirmed that D-apiose could serve as the sole carbon source to support the growth of strain LCG007 (Figure 4 and Supplementary Figure S3). Similarly, mannopine is a plant metabolite derived from D-mannitol (Kim et al., 2001; Swain et al., 2012), and we identified a strain-specific operon for the ABC transport system of mannopine (AB1M95_RS17500 to AB1M95_RS17515) in strain LCG007 (Supplementary Table S4). Putatively, these genes could assist strain LCG007 in better adapting to the nutritional conditions of the intertidal zone environment, where there is a substantial transport of terrigenous organic carbon.

It is worth noting that, compared to its possession of many genes encoding extracellular peptidases, only one extracellular glycoside hydrolase gene was predicted (belonging to the GH25 family), suggesting that its breakdown of sugars primarily occurs intracellularly. Indeed, in both strains LCG007 and D53, we identified a conserved gene operon involved in both starch synthesis and the hydrolysis of malto-oligosyltrehalose, which includes the genes of starch synthase (EC: 2.4.99.16), maltose α-D-glucosyltransferase (EC: 5.4.99.16), 1,4-α-glucan branching enzyme (EC: 2.4.1.18), a putative glycosyl hydrolase, malto-oligosyltrehalose trehalohydrolase (EC: 3.2.1.141), and (1–>4)-α-D-glucan 1-α-D-glucosylmutase (EC: 5.4.99.15). Putatively, the intake and intracellular transformation of complex carbohydrates into storage forms (e.g., branched starch) may endow strains LCG007 and D53 with a competitive edge in carbon acquisition, potentially enabling them to dominate in resource scarcity and sustain growth amid organic carbon limitations, thereby ensuring an advantageous ecological position.

#### 3.4.3 Organic acid metabolism

Strain LCG007 possessed 91 transporter genes for organic acids, including ABC transport systems for silicic acid, tripartite tricarboxylate, and C4-dicarboxylate, as well as formate/nitrite transporters, long-chain fatty acid transport proteins and AEC family malonate transporters. Among these transporters, about half (45 genes) exhibited no sequence similarity with other nine closely related strains, suggesting that horizontal gene transfer might significantly contribute to strain LCG007's capability to utilize organic acids.

We further showed that strain LCG007 is capable of utilizing lactic acid, malic acid, citric acid, β-hydroxy-D, L-butyric acid, bromo-succinic acid, acetoacetic acid through the Biolog GEN III system (Supplementary Table S6). Moreover, sodium malate, sodium benzoate, sodium citrate and sodium acetate could serve as the sole carbon source for its growth (Figure 4 and Supplementary Figure S3). Additionally, polyhydroxybutyrate (PHB), a common form of biologically stored carbon within many bacterial species, serves as an energy reserve material. Strain LCG007 possesses a gene encoding extracellular PHB depolymerase, and could grow with PHB as the sole carbon source (Figure 4 and Supplementary Figure S3). These pieces of evidence suggested that organic acid metabolism is also crucial for its survival.

#### 3.4.4 Nitrogen metabolism

In strain LCG007, we identified one gene encoding ammonium transporter. Moreover, it carries a set of assimilatory nitrate reductase gene (*nasC*) and nitrite reductase genes (*nasDE*) on its plasmid, which are immediately adjacent to the genes of nitrate/nitrite ABC transport system (*nrtABC*; Supplementary Table S4). It suggested its capability of utilizing both ammonia, nitrate and nitrite as nitrogen sources.

Moreover, its ability to utilize organic nitrogen is quite robust, as evidenced by abundant amino acid metabolic genes as mentioned above, as well as the presence of 10 gene operons encoding spermidine/putrescine ABC transport systems (e.g., *potABCD, potFGHI*; Supplementary Table S4). Polyamines in the marine environment primarily originate from the degradation of organic matter, such as the breakdown of phytoplankton and other marine organisms, which releases these compounds into the water column. Such capability could enable strain LCG007 to adapt to the nutritional conditions in surface seawater.

Besides, it has genes for a urea transporter (*urtABCDE*) and urease (*ureABCDEFG*) (Supplementary Table S4), and could grow with urea as the sole nitrogen source (Figure 4 and Supplementary Figure S3). Moreover, xanthine permease (*xanP*), and a general nucleoside ABC transport system (*nupABC*) were predicted in strain LCG007 (Supplementary Table S4). Unlike strain D53, strain LCG007 has gene encoding xanthine dehydrogenase, which enables it to degrade xanthine. Our experiments have also confirmed that it can grow on a medium with xanthine as the sole substrate (Figure 4 and Supplementary Figure S3). Such a broad nitrogen substrate utilization capacity collectively supports its growth and activity in this eutrophic but ever-changing intertidal habitats.

#### 3.4.5 Phosphorus and sulfur metabolism

As for phosphorus utilization, strain LCG007 is equipped with a comprehensive suite of phosphorus transporters, including two phosphonate-specific ABC transporters, alongside a phosphatephosphonate ABC transporter, a phosphate:Na^+^ symporter, and a PiT family phosphate transporter. Additionally, it also contains a gene encoding extracellular PhoX family phosphatase, which is capable of dephosphorylating various phosphor-containing compounds (Supplementary Table S4). Such abundant phosphorus metabolic capabilities may facilitate its survival in phosphorus-limited marine surface waters.

As for sulfur metabolism, strain LCG007 boasts a sophisticated enzymatic arsenal for sulfur metabolism. This includes the sulfite dehydrogenase (quinone) complex, which is encoded by the *soeABCD* and is instrumental in the oxidation of sulfite to sulfate, a critical step in the sulfur oxidation pathway (Supplementary Table S7). Additionally, the strain features the sulfur oxidation (SOX) complex, with genes *soxXYZABCD* responsible for the initial oxidation of inorganic sulfur compounds, particularly sulfide and thiosulfate, further highlighting its metabolic prowess in harnessing sulfur compounds (Supplementary Table S7).

Besides, strain LCG007 possesses the genes for the taurine ABC transport system (*tauABC*) and taurine-pyruvate aminotransferase (EC: 2.6.1.77), which is crucial for the uptake and metabolism of taurine, an important sulfur-containing amino acid derivative (Supplementary Table S7). Moreover, a strain-specific gene encoding taurine dioxygenase was found in the genome of strain LCG007, which typically involves the conversion of taurine to sulfoacetaldehyde and hydrogen peroxide as a byproduct. Additionally, strain LCG007 also has a gene of DMSP lyase (DddQ), which catalyzes the breakdown of DMSP into DMS, a volatile climate-active gas. The presence of these genes potentially offers insights into the metabolic capabilities and ecological roles of strain LCG007, particularly in sulfur cycling and detoxification processes.

### 3.5 Light enhancing growth under oligotrophic conditions

Many studies have reported that multiple RCA species possess the capability for aerobic anoxygenic photosynthesis (AAPS), which allows them to use organic matter, sulfides, or ammonia as hydrogen donors to capture light energy through bacterial chlorophyll under aerobic conditions for photosynthesis without releasing oxygen (Voget et al., 2015). In this study, we identified a 44.4-kb gene cluster in strain LCG007 (Supplementary Table S8), putatively involved in photosynthesis, including assembly of photosynthetic complexes, production of chlorophyll precursors, and synthesis of light-harvesting pigments (e.g., carotenoids and bacteriochlorophyll).

Moreover, our genomic comparison showed that these photosynthesis-related genes are highly conserved in strains of LCG007, D53, HSMS-29, and the six *Roseobacter* type strains (Supplementary Table S9). However, among the 12 type strains of the *Sulfitobacter* genus, only three have the capability for photosynthesis, and the strains containing these genes are not phylogenetically contiguous (Figure 2). This suggested that the acquisition of photosynthesis genes could be very important for the speciation of the common ancestor of genera *Roseobacter* and *Sulfitobacter_E*; whereas the photosynthetic ability in *Sulfitobacter* might have been acquired through horizontal gene transfer after this genus has formed.

**Figure 5:**
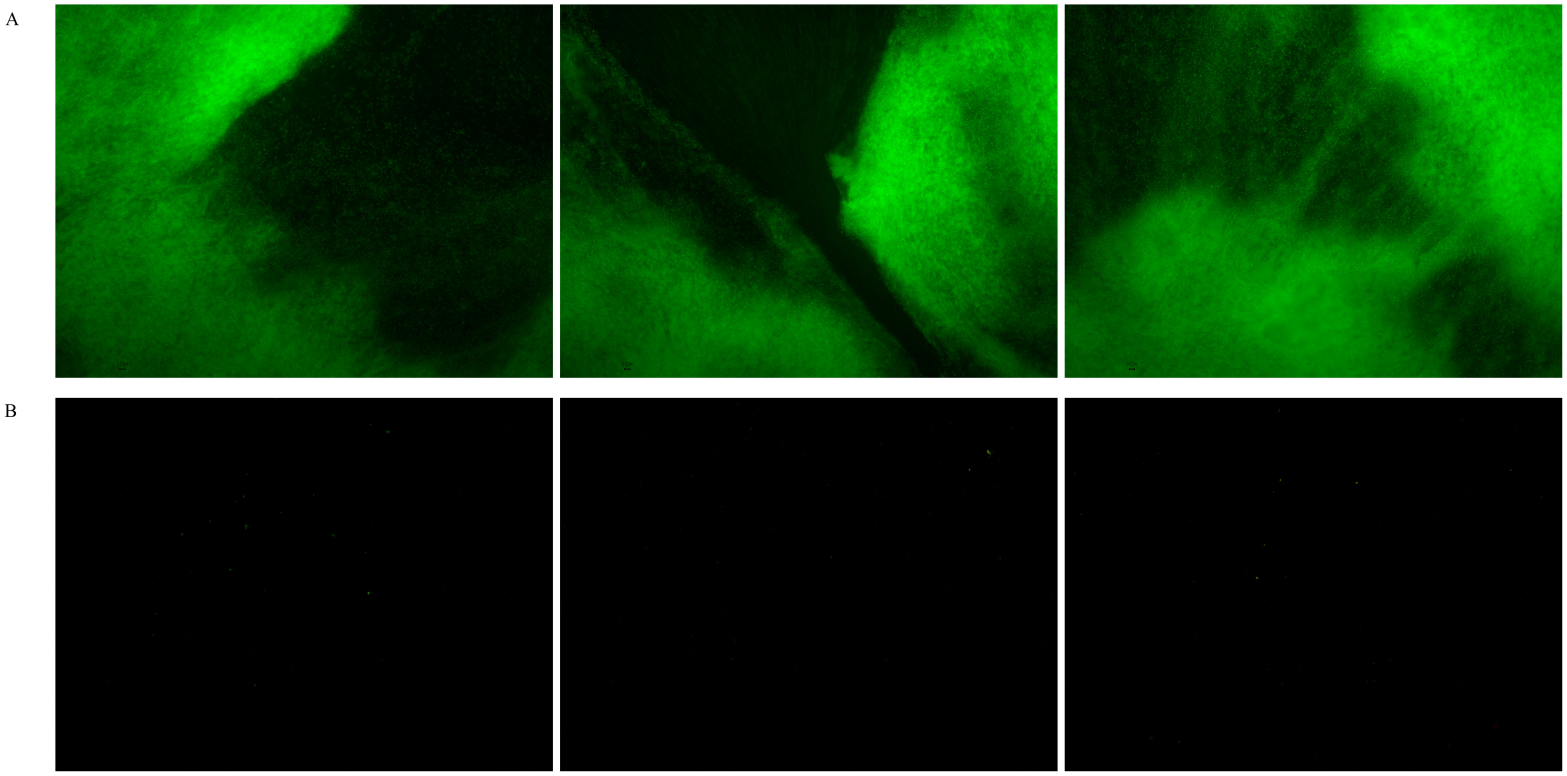
The influence of visible light on strain LCG007's morphology under oligotrophic conditions was investigated. Bacterial cultures were stained with fluorescent dyes and subsequently examined under a microscope after a 72-h incubation period. The samples were analyzed under two distinct conditions: exposure to visible light **(A)** and incubation in the dark **(B)**. Each experiment has three biological replicates.

Due to the current lack of clarity on the effect of visible light on the growth of *Sulfitobacter_E* species, we conducted cultivation experiments with strain LCG007 using a low-nutrient medium containing only 1% of the organic content from MB 2216E. Our results indicated that the group exposed to light had a significantly higher optical density (OD) than the group kept in the dark. Additionally, we observed a substantial amount of blocky material at the bottom of the culture containers. Further analysis using the SYTO 9/PI staining method revealed that these deposits were aggregation of living cells (Figure 5). These observations strongly suggested that light is a pivotal factor influencing the metabolism and physiology of strain LCG007, particularly under nutrient-limited conditions.

### 3.6 The adaptation mechanisms of strain LCG007 to intertidal environments

Intertidal microorganisms are continually subjected to the environmental vicissitudes of their habitat, including transient oxygen levels that precipitate the formation of reactive oxygen species (ROS), exemplified by superoxide anions and hydrogen peroxide. Concurrently, salinity and thermal oscillations can perturb cellular metabolism, thereby exacerbating endogenous ROS formation. Moreover, solar irradiation induces ROS generation via photochemical mechanisms. To counteract oxidative stress, strain LCG007 has developed a robust enzymatic defense system, featuring 10 peroxidases, three superoxide dismutases and one strain-specific catalase, which facilitate its survival in the intertidal zone (Supplementary Table S10).

The intertidal zone also experiences a dramatic range of temperatures, with diurnal and seasonal fluctuations, exposing organisms to a challenging thermal gradient. These variations are further intensified by the direct exposure to solar radiation during low tide and the cooling effects of submerged immersion during high tide. Heat shock proteins (HSPs) and cold shock proteins (CSPs) play crucial roles in temperature resistance by aiding in the refolding of denatured proteins and maintaining cellular homeostasis during thermal stress, respectively. We identified two HSPs and six CSPs, which facilitate the cellular response to temperature extremes by maintaining protein homeostasis and stabilizing nucleic acid structures (Supplementary Table S10).

Furthermore, during periods of low tide, microbes are exposed to air and consequently experience high levels of ultraviolet (UV) radiation, which often results in the formation of pyrimidine dimers. Strain LCG007 is equipped with a deoxyribodipyrimidine photo-lyase gene and a (6–4) DNA photolyase gene (EC:4.1.99.13, also known as *PhrB*), both of which facilitate the repair of pyrimidine dimers via the process of photoreactivation (Supplementary Table S10). This enzymatic activity putatively enables strain LCG007 to counteract the genotoxic effects of UV radiation, thereby ensuring its viability and metabolic activity in the harsh intertidal habitat.

The intertidal zone undergoes substantial salinity changes due to periodic high tides bringing in seawater and evaporation during low tides, creating a fluctuating environment that presents osmotic stress to its inhabitants. These salinity shifts can span from nearly freshwater to extremely saline conditions, necessitating strong adaptive mechanisms among intertidal organisms. Osmoprotectants, such as glycine betaine, proline, and trehalose, are small, neutral molecules that help maintain cellular homeostasis under osmotic stress. For strain LCG007, it possesses an ABC transporter for glycine betaine/proline and a BCCT transporter for betaine (Supplementary Table S4). Additionally, it has two adjacent genes that encode trehalose-phosphatase and trehalose-6-phosphate synthase, which may help maintain intracellular trehalose levels to adapt to the variable intertidal environment. Furthermore, strain LCG007 contains a strain-specific γ-aminobutyraldehyde dehydrogenase (EC:1.2.1.19, AB1M95_RS09460), implicated in the production of γ-aminobutyric acid (GABA). Some studies propose that GABA, acting as an osmoregulator, may be involved in microbial stress responses, aiding in preserving solute balance within the cell to counter external osmotic changes (Bhatt et al., 2024).

Interestingly, we observed that strain LCG007 robustly forms biofilm under high NaCl concentration (more than 3% w/v), regardless of the presence or absence of light (Supplementary Figure S4). We hypothesize that this pronounced biofilm formation is a strategic adaptation that serves multiple purposes. It not only provides a relatively stable microenvironment, shielding strain LCG007 from environmental fluctuations such as high salinity, desiccation, and temperature shifts, but also retains moisture within the extracellular polymeric substances (EPS). This moisture retention is essential for helping cells combat the dehydration caused by high osmotic pressure typically found in high salinity environments, such as the intertidal sediment surface after being exposed by the receding tide and then sunbaked.

**Figure 6:**
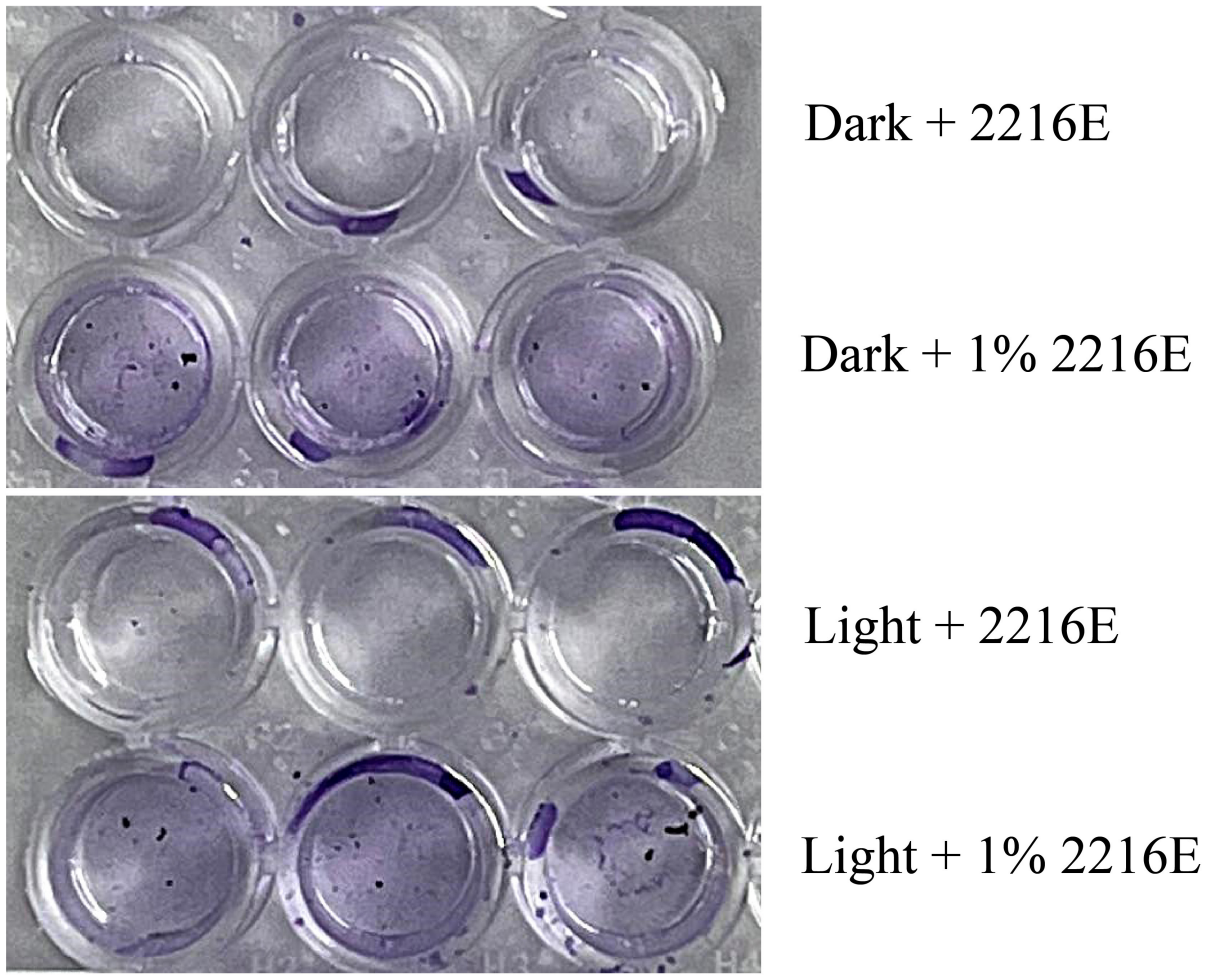
Experiment on the measurement of biofilm-forming properties of strain LCG007 under conditions of light/darkness and nutrient-rich/nutrient-poor environments. Each experiment has three biological replicates.

Additionally, nutrient substances in the intertidal zone are affected by tidal influences. We found that an oligotrophic medium (containing 1% of the organic components of MB 2216E) also promotes strain LCG007 to form more biofilm compared to a nutrient-rich medium (MB 2216E), and this is independent of whether there is light exposure or not (Figure 6). We proposed that, firstly, biofilms can provide strain LCG007 with a protective barrier that minimizes the impact of other adverse environmental stresses when facing oligotrophic conditions, desiccation, temperature fluctuations, and chemical pressures. Secondly, biofilms may facilitate the exchange of nutrients between strain LCG007 and other microorganisms, and enhance their ability to cooperatively metabolize certain compounds, thereby increasing the adaptability and survival capacity of the entire community.

## 4 Conclusions

In conclusion, strain LCG007 was isolated from the intertidal waters of Lu Chao Harbor. Phylogenetically, it could represent a novel genus within family Rhodobacteraceae, which is label as *Sulfitobacter_E* in GTDB taxonomy. Metabolically, it possesses genes for the intake and degradation of amino acids and peptides, including those containing D-amino acids, which are crucial for the cycling of refractory organic matter. The strain's ability to utilize a variety of carbohydrates including plant-derived sugars, along with its strain-specific capacity to utilize diverse phosphorous, sulfur and nitrogen sources, particularly polyamines, underscores its metabolic diversity. The widespread presence of photosynthesis gene clusters in *Sulfitobacter_E* and *Roseobacter* species, as opposed to their sporadic distribution in the neighboring *Sulfitobacter* genus, emphasizes that the acquisition of photosynthetic genes could play a crucial role in the speciation of the common ancestor of *Sulfitobacter_E* and *Roseobacter*, particularly in relation to their adaptation to marine surface habitats. The genetic repertoire that encodes DNA photolyase, heat/cold shock proteins, ROS-scavenging enzymes, and the machinery for the assimilation and production of osmoprotectants such as betaine, GABA, and trehalose, as well as the ability to form biofilms under oligotrophic or high-salinity conditions, collectively facilitate strain LCG007's adaptation to the dynamic intertidal environment. This research provides new insight into the metabolism and adaptive mechanisms of RCA cluster members in intertidal habitats, emphasizing their crucial part in marine nutrient cycling.

## Data Availability

The datasets presented in this study can be found in online repositories. The names of the repository/repositories and accession number(s) can be found below: https://www.ncbi.nlm.nih.gov/, GCF_040801785.1.
