# Supplementary material for: Phylogenetic analysis, metabolic profiling, and environmental adaptation of strain LCG007: a novel Rhodobacteraceae isolated from the East China Sea intertidal zone
Source: Front Microbiol. 2025 Jan 7;15:1533195. doi: 10.3389/fmicb.2024.1533195 (PMC11747546; doi:10.3389/fmicb.2024.1533195)

**Supplementary Tables and Figures Legends**

**Supplementary Tables Legends**

**Supplementary Table S1:**

The ANI values and aligned percentage between strain LCG007 and other strains.

**Supplementary Table S2:**

The AAI values and aligned percentage between strain LCG007 and other strains.

**Supplementary Table S3:**

The COG classification of strain LCG007.

**Supplementary Table S4:**

The transporters predicted in strain LCG007.

**Supplementary Table S5:**

The Extracellular enzymes predicted in strain LCG007.

**Supplementary Table S6:**

The Biolog GenIII test results of strain LCG007.

**Supplementary Table S7:**

The enzymatic arsenal for sulfur metabolism predicted in strain LCG007.

**Supplementary Table S8:**

The photosynthesis gene cluster comparison between strain LCG007 and other strains.

**Supplementary Table S9:**

The photosynthesis gene cluster predicted in strain LCG007.

**Supplementary Table S10:**

Prediction of genes related to the adaptation of strain LCG007 to intertidal environments.

**Supplementary Figures**

**Supplementary Figure S1**: The type of respiratory quinones of strains LCG007.


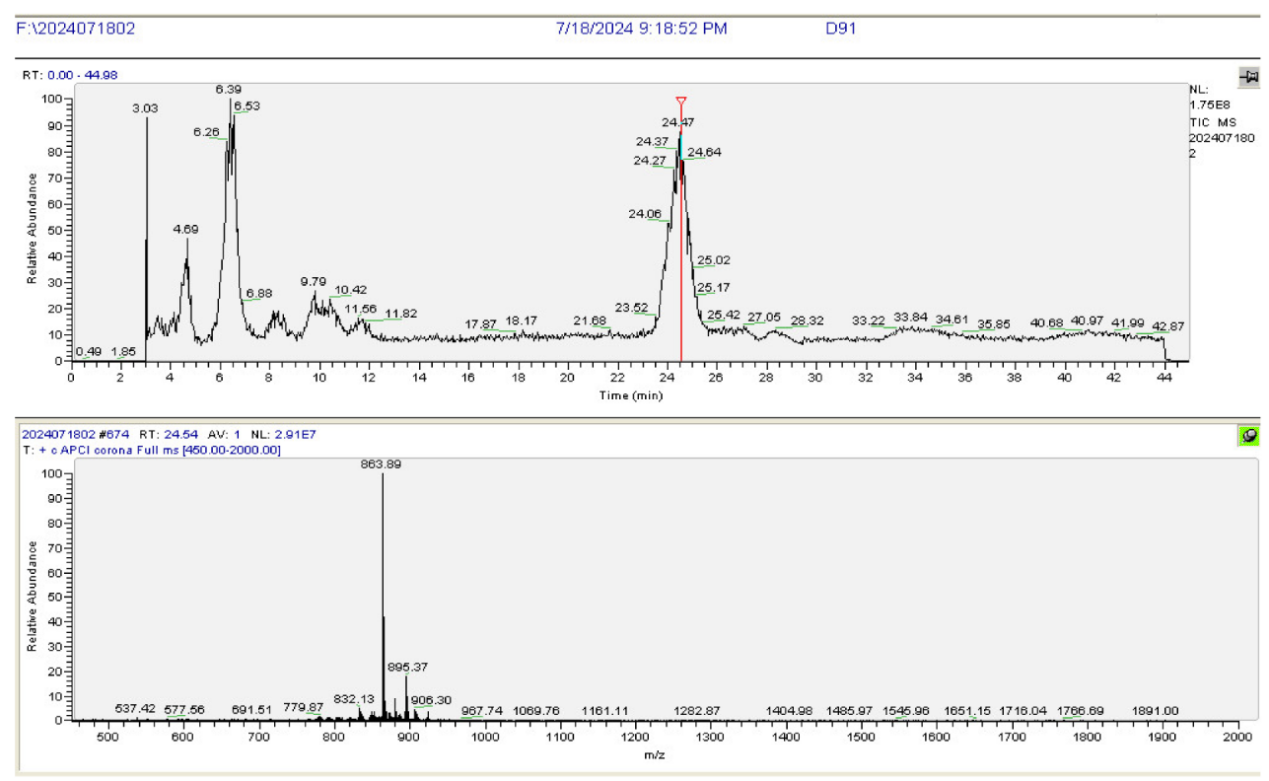


**Supplementary Figure S2**: The polar lipids of strain LCG007, total lipid (A), phospholipid (B), glycolipid (C), and aminolipid (D). Abbreviations: phosphatidylcholine (PC), phosphatidylethanolamine (PE), phosphatidylglycerol (PG), diphosphatidylglycerol (DPG), phosphoglycolipid (PGL), aminoglycophospholipid (APGL), aminolipids (AL), phospholipids (PL), glycolipids (GL), unidentified lipids (L).


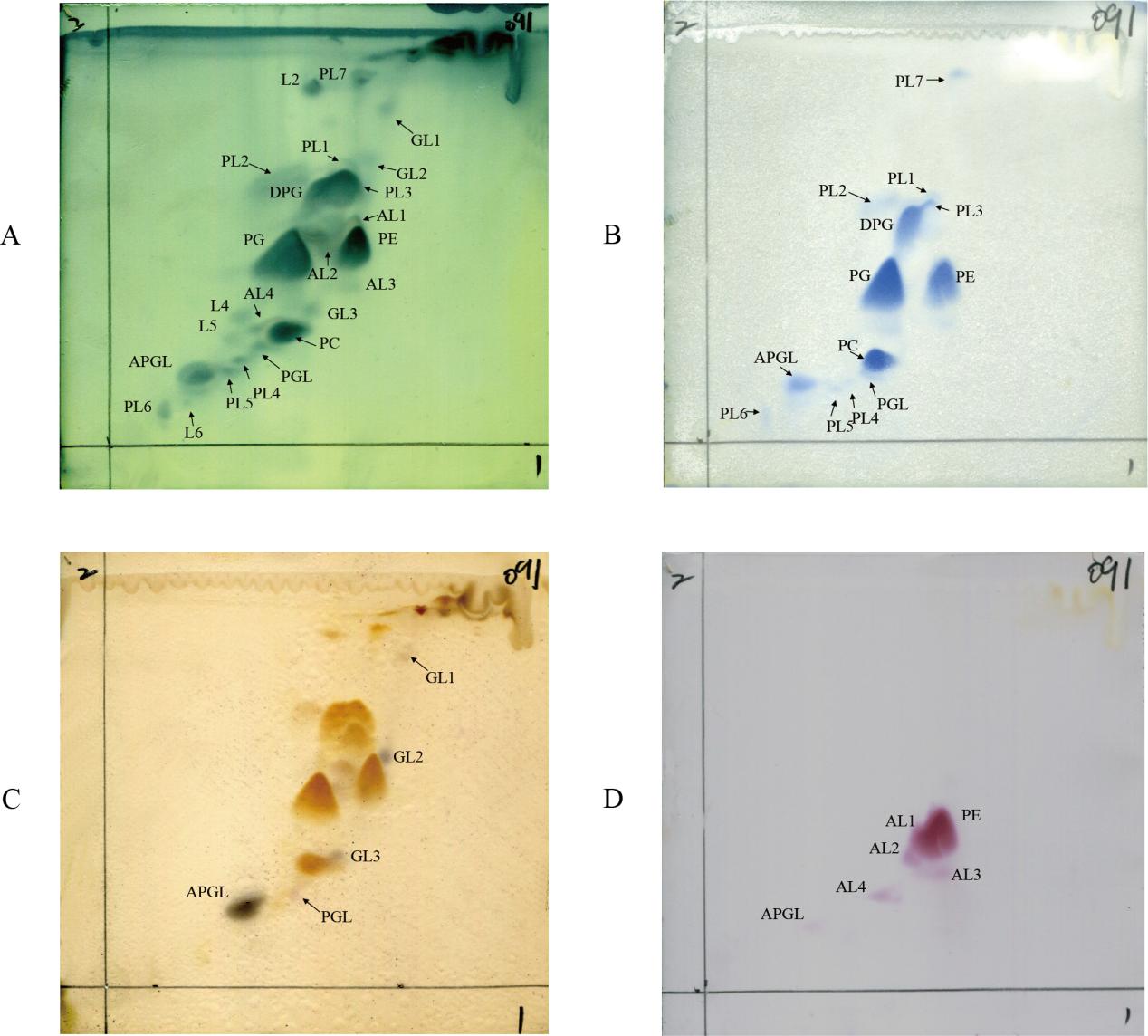


**Supplementary Figure S3**: The colony of strain LCG007 under different carbohydrates on MB 2216E agar plates. From A to P, each represents peptone, D-alanine, D-phenylalanine, D-lysine, D-glutamate, peptidoglycan, D-apiose, sodium malate, sodium benzoate, sodium citrate, sodium acetate, polyhydroxybutyrate, urea, hypoxanthine, xanthine, casein, tyrosine and blank. The subfigures in the left column depict 2216E agar plates inoculated with cultures that were not incubated (0 days), while those in the right column illustrate the plates with cultures incubated for 7 days. Each experiment was evaluated using three independent biological replicates.


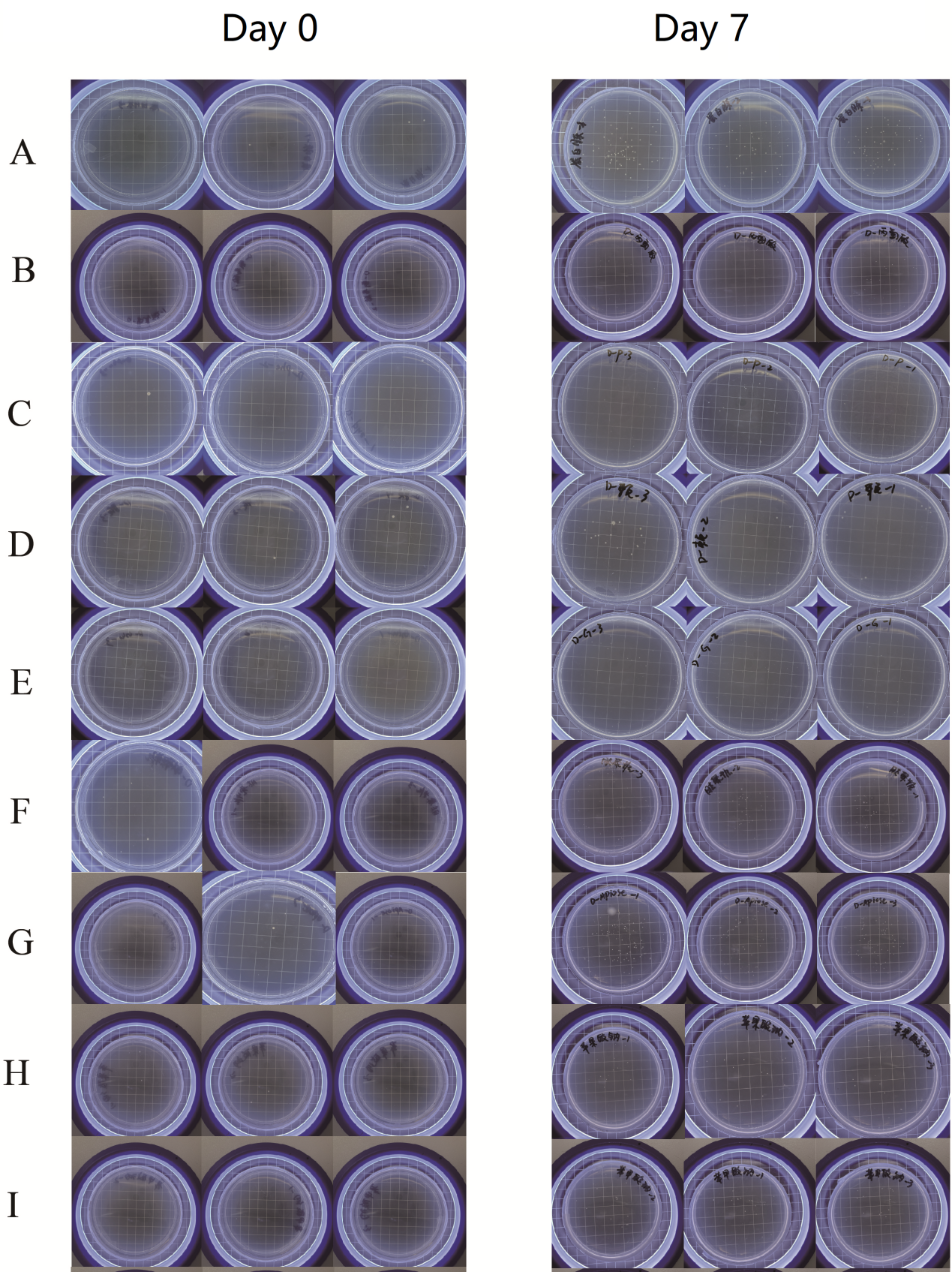


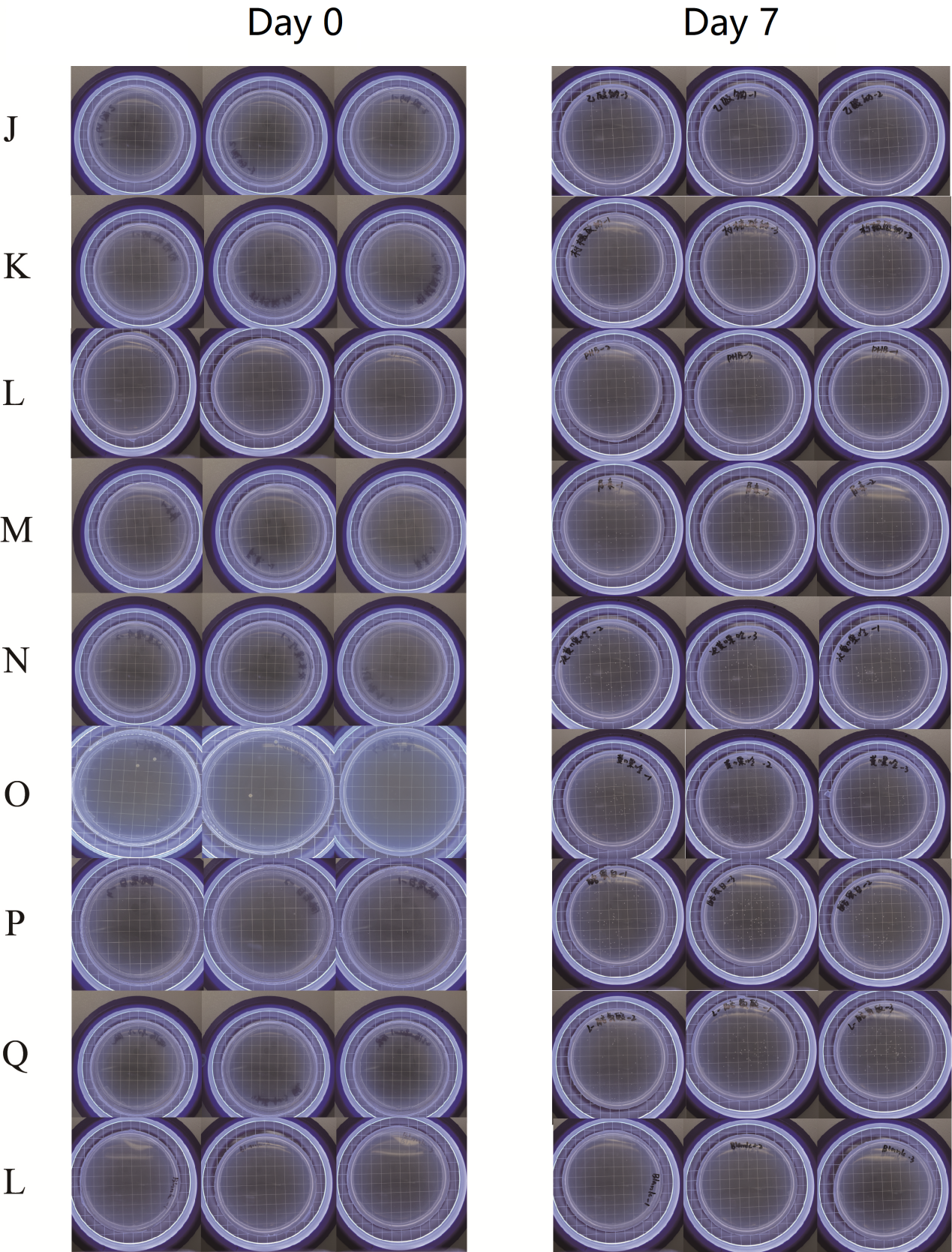


**Supplementary Figure S4**: The growth status of strain LCG007 under various salinities (MB 2216E). The NaCl concentrations for A through F are 0%, 1%, 2%, 3%, 4%, 5%, and 6%, respectively. Each experiment was evaluated using three independent biological replicates.


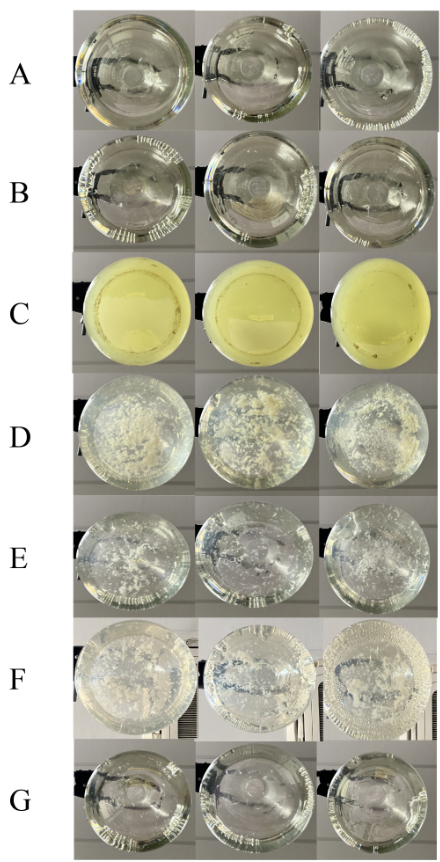

Supplement: Supplementary file 11 [file Data_Sheet_1.docx]
